# Detection of Alphacoronavirus vRNA in the Feces of Brazilian Free-Tailed Bats (*Tadarida brasiliensis*) from a Colony in Florida, USA

**DOI:** 10.3390/diseases5010007

**Published:** 2017-02-27

**Authors:** Tania S. Bonny, John P. Driver, Taylor Paisie, Marco Salemi, John Glenn Morris, Lisa A. Shender, Lisa Smith, Carolyn Enloe, Kevin Oxenrider, Jeffery A. Gore, Julia C. Loeb, Chang-Yu Wu, John A. Lednicky

**Affiliations:** 1 Department of Environmental and Global Health, University of Florida, Gainesville, FL 32610, USA; tbonny@phhp.ufl.edu (T.S.B.); jloeb@phhp.ufl.edu (J.C.L.); 2 Emerging Pathogens Institute, University of Florida, Gainesville, FL 32611, USA; tpaisie@ufl.edu (T.P.); salemi@pathology.ufl.edu (M.S.); jgmorris@epi.ufl.edu (J.G.M.Jr.); 3 Department of Animal Sciences, University of Florida, Gainesville, FL 32611-0910, USA; jdriver@ufl.edu; 4 Genetics and Genomics, Genetics Institute, University of Florida, Gainesville, FL 32610, USA; 5 Department of Pathology, Immunology and Laboratory Medicine, University of Florida, Gainesville, FL 32610, USA; 6 Department of Medicine, University of Florida, Gainesville, FL 32610-0277, USA; 7 Florida Fish and Wildlife Conservation Commission, Gainesville, FL 32601, USA; Lisa.Shender@MyFWC.com (L.A.S.); Lisa.Smith@myfwc.com (L.S.); Carolyn.Enloe@myfwc.com (C.E.); Kevin.J.Oxenrider@wv.gov (K.O.); Jeff.Gore@MyFWC.com (J.A.G.); 8 Department of Environmental Engineering Sciences, University of Florida, Gainesville, FL 32611, USA; cywu@essie.ufl.edu

**Keywords:** Brazilian free-tailed bats, alphacoronavirus, RNA-dependent RNA polymerase (RdRp) gene

## Abstract

Bats are natural reservoirs of coronaviruses and other viruses with zoonotic potential. Florida has indigenous non-migratory populations of Brazilian free-tailed bats (*Tadarida brasiliensis*) that mostly roost in colonies in artificial structures. Unlike their counterparts in Brazil and Mexico, the viruses harbored by the Florida bats have been underexplored. We report the detection of an alphacoronavirus RNA-dependent RNA polymerase (RdRp) gene sequence in the feces of two of 19 different *T. brasiliensis* that were capture/release bats that had been evaluated for overall health. The RdRp sequence is similar but not identical to previously detected sequences in the feces of two different species of bats (*T. brasiliensis* and *Molossus molossus*) in Brazil. In common with the experience of others doing similar work, attempts to isolate the virus in cell cultures were unsuccessful. We surmise that this and highly related alphacoronavirus are carried by Brazilian free-tailed bats living in a wide eco-spatial region. As various coronaviruses (CoVs) that affect humans emerged from bats, our study raises the question whether CoVs such as the one detected in our work are yet-to-be-detected pathogens of humans and animals other than bats.

## 1. Introduction

Bats (order *Chiroptera*, suborders *Megachiroptera* and *Microchiroptera*) are a widely distributed group of mammals that comprise ~20% of all known mammalian species [1]. They are reservoirs of many emerging and reemerging zoonotic viruses, some of which are highly pathogenic in humans. The emerging viruses exert a significant public health threat [2,3] and include ebolaviruses, henipaviruses, lyssaviruses and coronaviruses [4,5,6,7,8,9]. These are all viruses that can cause infections through inhalation routes of exposure, and viruses such as *Hendra*, *Nipah*, and SARS viruses cause severe respiratory infections in humans.

Coronaviruses, order *Nidovirales*, family *Coronaviridae*, subfamily *Coronavirinae*, are enveloped positive-sense single-stranded RNA viruses. There are four CoV genera: *Alphacoronavirus*, *Betacoronavirus*, *Gammacoronavirus* and *Deltacoronavirus* [10]. After it was found that SARS-CoV probably originated in bats [11,12], a flurry of investigations uncovered many more novel bat CoVs [13,14,15,16,17,18,19,20,21,22,23,24,25,26]. The recent description of a bat CoV related to MERS-CoV in Mexican bats [27] emphasized the relevance of investigating neotropical bats for CoVs.

Thirteen different species of insectivorous bats are found in Florida [28]. Brazilian free-tailed bats (*Tadarida brasiliensis*), also known as Mexican free-tailed bats, are one of the most abundant species of bats found throughout Florida, except the Florida Keys [28]. They roost in large colonies and, in Florida, they roost mostly in man-made structures, including buildings and bridges [28]. Despite the abundance and potential role of bats in disease transmission, viruses harbored by Florida bats remain mostly underexplored. With human activity increasingly overlapping the habitats of bats, the possibility of disease outbreaks resulting from spillover of bat CoVs cannot be ruled out [29]. Although no human diseases caused by a bat CoV have been identified in Florida, surveillance of CoVs in bat species is necessary to better predict and prevent the next emergence of a CoV disease outbreak [29].

In this study, we investigated whether CoV vRNA could be detected in the feces of Brazilian free-tailed bats in Florida.

## 2. Materials and Methods

For this study, free-tailed bats were chosen for two reasons: (a) opportunity; the bats were from a conservation site wherein the animals’ well-being is periodically evaluated and bat fecal samples were available for evaluation; and (b) they are among the most abundant bats often found roosting in buildings in Florida, and hence are most likely to interact with humans. These bat species were identified and evaluated for overall health by expert bat biologists of the Florida Fish and Wildlife Conservation Commission (FWC). The FWC has no designated or required IACUC protocol, however they follow the guidelines of American Society of Mammalogists for the capture and handling bats [30]. Nineteen (*n* = 19) fecal samples were collected from capture/release bats in Gilchrist County, 8 km southwest of Ft. White, Florida in May 2016. Following collection, the samples were immediately sent to a BSL2-enhanced laboratory and stored at −80 °C. Bat fecal pellets were homogenized to 10% (*w*/*v*) suspensions in Gibco™ advanced Dulbecco’s Modified Eagle Medium (aDMEM) (Fisher Scientific, Pittsburgh, PA, USA, Cat#12491015) supplemented with 0.2 mM glutamine (Gibco™ GlutaMAX, Fisher Scientific, Cat# 35050-061), antibiotics (50 μg/mL penicillin, 50 μg/mL streptomycin, 100 μg/mL neomycin (PSN, Fisher Scientific, Cat #15640055)) using Covidien Precision™ disposable tissue grinders (Fisher Scientific, Cat# 06-434-1). The homogenates were cleared of debris by low-speed centrifugation (5 min at 1500× *g*), and the supernatants filtered through 0.45 μm PVDF, sterile filters (Fisher Scientific, Cat# 09-720-4) to remove bacteria and other particulates, and the filtrates stored at −80 °C until further use.

American Type Culture Collection (ATCC, Manassas, VA, USA) cell lines VERO E6 (African green monkey kidney; CRL-1586), A549 (human lung adenocarcinoma epithelium; CCL-185), and Tb1 Lu (*Tadarida brasiliensis* lung epithelium; CCL-88) were propagated as monolayers as previously described [31], and a newly confluent monolayer of each of these three cell lines was inoculated with aliquots (75 μL) of the filtered homogenates. The inoculated cells were incubated in a humidified 5% CO_2_ atmosphere at 35 °C, and observed daily for virus-specific cytopathic effects (CPE).

Viral nucleic acids were extracted from both filtered homogenates and spent cell media using the QIAamp viral RNA minikit (Qiagen, Germantown, MD, USA, Cat#52904). CoV RNA screening was performed by reverse transcription-polymerase chain reaction (RT-PCR) targeting conserved region of the RNA-dependent RNA polymerase (RdRp) gene. Briefly, viral RNA was denatured at 65 °C for 5 min in the presence of SUPERase-In RNase inhibitor (Invitrogen Corp., Carlsbad, CA, USA, Cat#AM2694), cooled rapidly on ice and cDNA synthesis performed with Omniscript Reverse Transcriptase (RT) (Qiagen, Cat# 205111) for 1 h at 37 °C using primer CorTheoNL63R1 (5′-CCRTCATCAGANAGAATCATCAT-3′). PCR was performed using One Taq DNA polymerase (New England BioLabs, Ipswich, MA, USA, Cat# M0480) with primer pair CorTheoNL63F1 (5′-GGTTGGGAYTATCCYAANTGTGA-3′) and CorTheoNL63R1. With an expected product size of 440 bp, PCR was performed as: initial denaturation step (94 °C for 2 min); followed by 40 cycles of 94 °C (60 s), 48 °C (60 s), 68 °C (60 s), and a final extension step at 68 °C for 5 min. PCR products were visualized by gel electrophoresis in a 1.5% ethidium bromide-stained agarose gel.

In preparation for sequencing, samples wherein a 440 bp PCR amplicon were initially observed were re-amplified using high-fidelity polymerases. Briefly, cDNA was produced using AccuScript High Fidelity Reverse Transcriptase (Agilent Technologies, Inc., Santa Clara, CA, USA, Cat# 200820) in the presence of SUPERase-In RNase inhibitor, and PCR was performed using Phusion Polymerase (New England BioLabs, Cat# M0530S) with denaturation steps performed at 98 °C. The re-amplified samples were individually electrophoresed in a 1.5% ethidium bromide-stained agarose gel and the 440 bp amplicon excised and purified using a Qiagen MinElute Gel Extraction kit (Qiagen, Cat# 28604). The purified 440 bp PCR amplicons were then subjected to Sanger Sequencing. Preliminary sequence analyses were performed with the NCBI BLAST software. For phylogenetic analyses, all available RdRp CoV sequences were downloaded from NCBI (http://www.ncbi.nlm.nih.gov/). The sequences were aligned using Clustal Omega [32] and manually edited in Bioedit [33]. Phylogenetic signal was investigated by likelihood mapping in the program TREE-PUZZLE [34] in order to assess the phylogenetic signal in the sequence alignment and to remove the appropriate identical sequences. The maximum likelihood tree was estimated using the best nucleotide substitution model (TPM3 + I + G4) according to the results from IQ-TREE [35]. Bootstrapping (1000 replicates) was also performed using the IQ-TREE software. This was done in order to statistically analyze branch support in the maximum likelihood tree. The maximum likelihood tree was then manually edited in FigTree (http://tree.bio.ed.ac.uk/software/figtree/) to display geographical locations of the sequences and to show branches with strong statistical support (bootstrap values greater than 95%).

## 3. Results

Virus-induced CPE were not observed in cell cultures during a four-week observation period, suggesting a virus(es) had not been isolated. However, CoVs do not always cause easily discernable CPE in the cell lines used for this study, so for additional evidence of virus isolation, RT-PCR tests were performed. Coronavirus RNAs were also not detected by RT-PCR of spent cell culture media collected and tested by RT-PCR every five days, and in RNA purified from the infected cells at the terminal observation time-point (30 days post-infection). Attempts to isolate CoVs from the inoculated cell lines were thus considered unsuccessful.

**Figure 1:**
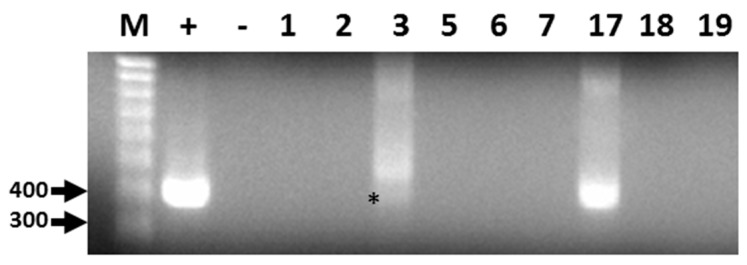
Representative results of RT-PCR detection of alphacoronavirus vRNA in Brazilian free-tailed bat feces (BF). Lane 1 (M), 100 bp MW markers; Lane 2 (+), HCoV-NL63 vRNA, positive control; Lane 3 (−), negative control; Lane 4, BF#1; Lane 5, BF#2; Lane 6, BF#3; Lane 7, BF#5; Lane 8, BF#6; Lane 9, BF#7; Lane 10, BF#17; Lane 11, BF# 18; Lane 12, BF#19. Virus-specific 440-bp PCR products amplified by PCR primers CorTheoNL63F1 and CorTheoNL63R1 are present in lanes 2, 6 (asterisk), and 10.

Out of 19 bat fecal samples, 440 bp amplicons corresponding to a conserved region of the CoV RdRp gene were generated by RT-PCR from two filtered homogenates (Figure 1). The sequence for both amplicons was identical and submitted to GenBank (Accession: KX663833.1). Following BLAST analyses, the consensus RdRp sequence was found to be highly similar but not identical to alphacoronavirus RdRp sequences identified in Brazilian free-tailed bats and velvety free-tailed bats (*Molossus molossus*) from southern Brazil [36]. The percentage of nucleotide and amino acid sequence identity ranged from 94% to 96%.

**Figure 2:**
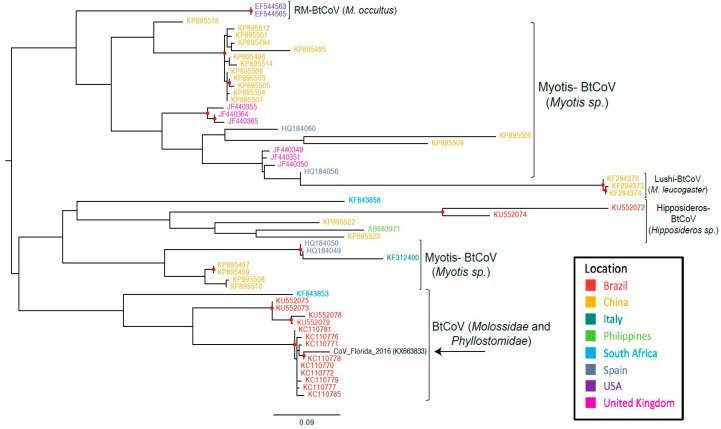
Maximum likelihood tree based on the nucleotide sequences of partial RdRp gene of bat CoVs. In parenthesis are the bat species that make up the clade. Abbreviations: BtCoV, bat coronavirus; Rm-BtCoV, Rocky Mountain bat coronavirus. Red circle indicates strong statistical support (bootstrap > 95%).

Phylogenetic analyses suggest that the RdRp gene sequence that had been RT-PCR-amplified from the feces of free-tailed bats in Florida clusters with RdRp gene sequences that were from two different types of bats in Brazil (Figure 2).

## 4. Discussion

Bats have been recognized as the natural reservoirs of a wide variety of viruses, many of which are important human and animal pathogens. Special attention has been paid to bat coronaviruses (BtCoVs) as the two emerging CoVs (SARS-CoV and MERS-CoV) causing human disease outbreaks in recent years are suggested to have emerged from bats [29]. It is plausible that other emerging BtCoVs may be able to cross the species barrier and cause human disease [29].

In Florida, 13 different species of insectivorous bats reside, 12 of which are year-round and only one species is seasonal [28]. Brazilian free-tailed bats are one of the permanent residents. Considering the potential public health implications of bat species living in close proximity to human inhabitants, the viruses harbored by these wide varieties of bat species in Florida have largely been underexplored. To our knowledge, this is the first report of alphacoronavirus vRNA detection in feces from presumably healthy insectivorous bats in Florida. The high degree of sequence similarity of our Florida BtCoV with that of a Brazilian BtCoV from two different bat species (*T. brasiliensis* and *M. molossus*) [36] suggests that similar CoVs may be present in different bat species and across geographically distant regions. Unlike the clade containing the Brazilian and Florida free-tailed bat CoVs, most of the other RdRp sequences cluster according to bat species, indicating that the viruses evolve according to bat species (Figure 2). In the other bat coronavirus clades, geographical location also appears to have an influence on the evolution of the viruses, but the clade with our sequence of interest from Florida contains bat species from the families *Molossidae* and *Phyllostomidae*. The similarity between our Florida BtCoV sequence and those of the Brazilian BtCoVs could be an indicator of how bat BtCoVs in Florida will evolve. The branch length in the maximum likelihood tree implies that the Florida BtCoV is diverging away from the Brazilian BtCoVs (Figure 2). The divergence of the Florida BtCoV could indicate the beginning of a new clade based on geographical location and not bat species. It is too early to infer other conclusions: Brazilian free-tailed bats are also found in Mexico and in Texas, where they are called Mexican free-tailed bats. Unfortunately, we were unable to find RdRp sequences for the BtCoVs of those bats in public databases, and it is plausible that those bat populations found geographically closer to Florida will harbor CoVs more similar to the one detected in our work.

Although restricted in sample number, location and the single bat species investigated, our study suggests that surveillance and identification of CoVs in Florida bats is worthy.
